# A worldwide bibliometric analysis of published literature on workplace violence in healthcare personnel

**DOI:** 10.1371/journal.pone.0242781

**Published:** 2020-11-23

**Authors:** Jesús Cebrino, Silvia Portero de la Cruz

**Affiliations:** 1 Department of Preventive Medicine and Public Health, Faculty of Medicine, University of Seville, Seville, Spain; 2 Department of Nursing, Pharmacology and Physiotherapy, Faculty of Medicine and Nursing, University of Córdoba, Córdoba, Spain; University of California San Diego, UNITED STATES

## Abstract

**Background:**

Workplace violence in healthcare professionals has become a worldwide public health problem and has been the focus of numerous publications; however, currently, no macroscopic overviews of this research based on bibliometric analysis have been carried out. Therefore, the main aim of this study was to analyse the research trends focusing on workplace violence in healthcare personnel over the last 27 years.

**Materials and methods:**

A bibliometric study was conducted from 1992 to 2019 in the field of workplace violence in healthcare personnel using the Scopus database. The author co-citation analysis was carried out using VOSviewer software. A worldwide map was created with Mapchart and word cloud image was created using Wordart. Descriptive and inferential statistics were applied.

**Findings:**

1791 records were analysed, 1376 of which (76.83%) were articles, with “*Medicine*” the most frequent subject category (58.91%). English was the predominant language (93.41%). From 2004 onwards, there was an exponential rise in the number of publications (R^2^ coefficient = 0.89; *p* < 0.0001) and the number of annual citations gradually increased from 1995 (R^2^ coefficient = 0.73; *p* < 0.0001). The University of Cincinnati (United States) was the institution (and country) with the highest number of publications (n = 30; n = 549), with D. M. Gates leading the ranking of the most productive authors (n = 21). *Journal of Nursing Management* was the most active journal publishing on the topic (n = 34) and the commonest keyword was “*human*/*s*” (16.43%).

**Conclusion:**

From 1992 to 2019, worldwide research into the published literature on workplace violence in healthcare personnel has grown steadily year by year, both in the number of documents and the number of citations. United States and their institutions and researchers dominates this research output.

## Introduction

Researchers currently have access to a large, fast-growing body of academic literature [1], and in this paper, bibliometric analysis has been applied to the topic of workplace violence in healthcare personnel. Nowadays, workplace violence against healthcare professionals is a global concern [2, 3], targeted at the very people who play a central role in making a healthcare system work, providing the population with health services and improving health outcomes [4]. The National Institute for Occupational Safety and Health [5] defines workplace violence as “*violent acts (including physical assaults and threats of assaults) directed toward persons at work or on duty*”. It is estimated that about one third of health workers are exposed to verbal abuse, sexual harassment, physical assault, aggression and threats from patients and visitors [6, 7].

Workplace violence has multiple negative physical [8] and psychological consequences for healthcare workers [9], leading to reduced job motivation [10], burnout [11], depression [12] and a desire to quit the job [13]. These consequences in turn affect the quality of care and put health-care provision at risk [14], not to mention the rise in absenteeism in the workplace [15] or increased costs, e.g. metal detectors and security guards [16].

The number of studies on workplace violence has risen substantially in recent years, there is still a need for a review of research patterns, as well as trends in health workers [17]. Bibliometric analysis is used to study a number of different indicators which allows to compare a variety of bibliometric statistics and correlations. Thus, the information presented in this article remains the best approach to providing provides a clear picture on the research progress achieved in violence on healthcare personnel, and it can assist practitioners and researchers in identifying fundamental influences from authors, journals, countries, institutions and keywords. This quantitative metric in conjunction with other types of metrics considered in our study and reflect the level of response elicited in the academic community (citation counts or h-index) serve as tools to assess research productivity [18]. On the other hand, it is important to take into account the output at the country level in the field of workplace violence in healthcare personnel because it provides a simple, objective measure (i.e. verifiable by anyone) of research performance, allowing cross-country comparisons to be made. In addition, the contribution from different countries is used by politicians, media and evaluation agencies when assessing scientific activity [19].

Bibliometric analysis provides a useful tool to study the development of global trends and offers an overview of the large number of publications, providing substantial empirical evidence to allow us to assess the impact of research knowledge on health issues [20]. It shows the latest advances, main topics, current gaps and cooperation patterns of researchers in a certain research field [7, 21]. At last, bibliometric analysis are nowadays abundantly used to inform research-policy and management decisions [22], for example, about research funding [23]. The main goal of this study is therefore to analyse the research trends focused on workplace violence in healthcare personnel over the last 27 years (from 1992 to 2019).

## Material and methods

The research strategy showed different results for Web of Science (1977 documents), Scopus (1823 documents), PubMed (1030 documents), the Health and Medical Collection (683 documents), and the Psychology Database (133 documents). These results displayed a similar number of documents for Web of Science and Scopus, lower number of documents for Pubmed, and the Health and Medical Collection and Psychology Database had the lowest quantity and also included greater number of grey data than the other three databases (Web of Science, Scopus and PubMed). The significant difference between these databases was the theme and topic of the research, therefore, the main two databases were Web of Science and Scopus. Only one database was selected based on the coverage of the topic and the objective, and the fact that previous research indicated how Web of Science and Scopus have high similarities [24]. Based on previous research and the results obtained, it was decided to choose Scopus (Elsevier’s database) since it was the major database focused on the topic [25, 26], providing necessary information for the quantitative analysis.

The literature search from 1992 to 2019 was performed on 29 January 2020 using the Scopus database. Scopus was used for this bibliometric content study because it is the largest abstract citation database of peer-reviewed literature, featuring smart tools to track, analyse and visualize research from over 23,500 journals (mostly peer-reviewed journals) and 194,000 books, as well as 9 million conference papers [27]. In addition, the Scopus database provides the most comprehensive overview of the world’s research output in numerous fields of knowledge [22, 28].

A sound search strategy is a key requisite for a successful search for a comprehensive set of documents on a study topic [29]. The search formula was defined as in Table 1. The field code “*TITLE-ABS-KEY*” was used in the formula, so that if the keywords were present in the title, abstract or keywords of any documents, the related publications would be shown in the findings. The literature from the Scopus database was retrieved using a set of search terms, focusing on (a) “*workplace violence*” and (b) “*healthcare personnel*”. The synonyms for these search terms were defined clearly and connected using the “*OR*” operator. Queries (a) and (b) were connected with the “*AND*” operator. The “*NOT*” operator was used to exclude records related to “domestic violence”.

**Table 1:** Search strategy for workplace violence in healthcare personnel.

Scopus (29/01/2020)		
Number	Concept	Search strategy
#1	Workplace violence	“Occupational violen*" OR "occupational agress*" OR "*occupational bullying*" OR “*occupational intimidation*” OR "occupational disturb*" OR "occupational pester*" OR "occupational sexual harass*" OR "occupational harass*" OR "occupational abus*" OR "occupational hostil*" OR "occupational offen*" OR "occupational extort*" OR "occupational threat*" OR "occupational assault*" OR "workplace violen*" OR "workplace agress*" OR "*workplace bullying*" OR “*workplace intimidation*” OR "workplace disturb*" OR "workplace pester*" OR "workplace sexual harass*" OR "workplace harass*" OR "workplace abus*" OR "workplace hostil*" OR "workplace offen*" OR "workplace extort*" OR "workplace threat*" OR "workplace assault*" OR “work-related violen*" OR "work-related agress*" OR "*work-related bullying*" OR “*work-related intimidation*” OR "work-related disturb*" OR "work-related pester*" OR "work-related sexual harass*" OR "work-related harass*" OR "work-related abus*" OR "work-related hostil*" OR "work-related offen*" OR "work-related extort*" OR "work-related threat*" OR "work-related assault*".
#2	Healthcare personnel	"Health care practitioner*" OR "health care assistant*" OR "*health care personnel*" OR "*health care staff*" OR "health care worker*" OR "health care professional*" OR "health care provider*" OR "healthcare practitioner*" OR "healthcare assistant*" OR "*healthcare personnel*" OR "*healthcare staff*" OR "healthcare worker*" OR "healthcare professional*" OR "healthcare provider*" OR "health practitioner*" OR "health assistant*" OR "*health personnel*" OR "*health staff*" OR "health worker*" OR "health professional*" OR "health provider*" OR "medical practitioner*" OR "medical assistant*" OR "*medical personnel*" OR "*medical staff*" OR "medical worker*" OR "medical professional*" OR "medical provider*" OR “midwi*” OR "nursing assistant*" OR "*nursing personnel*" OR "nursing professional*" OR "nursing provider*" OR "*nursing staff*" OR "nursing worker*" OR "nursing practitioner*" OR “*nurse*” OR “*nurses*” OR "physician*" OR "doctor*" OR "clinician*" OR "dentist*" OR "dental assistant*" OR "*dental personnel*" OR "*dental staff*" OR "dental worker*" OR "dental provider*" OR "dental professional*" OR “dental practitioner*” OR "pharmacist*" OR "physical therapist*" OR "physiotherapist*" OR "physical therapist assistant*" OR "*allied health personnel*" OR "allied health professional*" OR "*allied health staff*" OR "allied health provider*" OR "allied health worker*" OR “allied health practitioner*” OR "paramedic*" OR "*paramedical personnel*" OR "paramedical professional*" OR "*paramedical staff*" OR "paramedical provider*" OR "paramedical worker*" OR "health manager*" OR "health care manager*" OR "healthcare manager*" OR "clinical officer*".
#3	Domestic violence	"*Spousal abuse*" OR "*spousal violence*" OR "*dating violence*" OR "*intimate partner violence*" OR "*intimate partner abuse*" OR "*domestic violence*" OR "*partner abuse*" OR "*gender-based violence*" OR "*GBV*".
Search strategy		#1 AND #2 NOT #3

Two researchers (JC and SPC) independently verified the data entry and collection. Publications for which full text was not available were excluded. The data were organized by document types, subject categories, languages, number of publications per year, number of citations per year, journal name(s), author name(s), author affiliation(s), countries, publication title(s), number of citations per publication, citations per year and keywords. The keywords included in this study were the author’s keywords, not the MeSH terms. We collected the impact factor and quartiles of journals in the 2018 Journal Citation Reports (JCR) and 2018 SCImago Journal Rank (SJR). The differences between the two researchers’ verifications were discussed and a consensus was then reached.

The author co-citation analysis was carried out using VOSviewer software (version 1.6.8, Center for Science and Technology, Leiden University, the Netherlands). This open-source program allows us to visualise bibliometric maps and identify networks importing datasets from several sources, including Scopus [30], where the records are saved under the name “*scopus*.*csv*”. Author co-citation analysis detects the intellectual structure of a research topic and is used to identify which authors are most frequently cited together. This analysis considers that two authors cited together share a thematic similarity, and a higher frequency of author co-citation implies a greater affinity between them [31]. Names of the authors have been standardized to avoid duplications.

The worldwide map was created with Mapchart (https://mapchart.net/world.html). Finally, a word cloud image was created, including all the keywords of the records as a visual semantic network using Wordart (https://wordart.com/). The larger the size of the keywords, the higher the frequency in the documents. Repeated keywords and the following terms were removed: article, controlled study, cross-sectional study(ies), major clinical study, prevalence, priority journal, questionnaire and statistic and numerical data, as they were not considered relevant to this analysis.

We applied descriptive statistical analysis using frequencies for document types, subject categories, languages, number of publications per year, number of citations per year, journal name(s), author name(s), author affiliation(s), countries, publication title(s) and keywords. The number of citations per publication (CPP) was expressed by mean and standard deviation. For this analysis, the software G-Stat version 2 (GlaxoSmithKline S. A., Madrid, Spain) was used. The graphs were created using Microsoft Excel 2016.

## Results

The total number of publications analysed in this study was 1791 for the period of 1992–2019. No records were obtained before 1992.

### Document type, subject categories and language of publication

As regards the document type, the majority were articles (n = 1,376; 76.83%), followed by reviews (n = 170; 9.49%). The number of letters (n = 66; 3.69%), notes (n = 66; 3.69%) and editorials (n = 56; 3.13%) was below 100. Finally, other document types such as short surveys (n = 18; 1.01%), conference papers (n = 15; 0.84%), book chapters (n = 11; 0.61%), books (n = 9; 0.50%) or *errata* (n = 4; 0.22%) amounted to less than 20 publications.

Table 2 shows the distribution of the subject categories. The thematic area with the highest percentage of documents was “*Medicine*” (58.91%), followed by “*Nursing*” (38.86%). These, together with “*Social sciences*” (7.98%) and “*Psychology*” (6.20%), were the only subject areas which exceeded 100 documents. The other categories were less common in the Scopus database.

**Table 2:** Subject categories focusing on workplace violence in healthcare personnel (1992–2019).

Subject area	Frequencies (n)	Percentages (%)
**Medicine**	1,055	58.91%
**Nursing**	696	38.86%
**Social sciences**	143	7.98%
**Psychology**	111	6.20%
**Business, management and accounting**	36	2.01%
**Environmental science**	30	1.68%
**Health professions**	30	1.68%
**Biochemistry, genetics and molecular biology**	29	1.62%
**Pharmacology, toxicology and pharmaceutics**	22	1.23%
**Engineering**	20	1.12%
**Agricultural and biological sciences**	15	0.84%
**Multidisciplinary**	14	0.78%
**Arts and humanities**	13	0.73%
**Economics, econometrics and finance**	8	0.45%
**Neuroscience**	8	0.45%
**Computer science**	5	0.28%
**Immunology and microbiology**	5	0.28%
**Chemical engineering**	4	0.22%
**Dentistry**	4	0.22%
**Decision sciences**	2	0.11%
**Mathematics**	2	0.11%
**Veterinary**	1	0.06%

**Fig 1:**
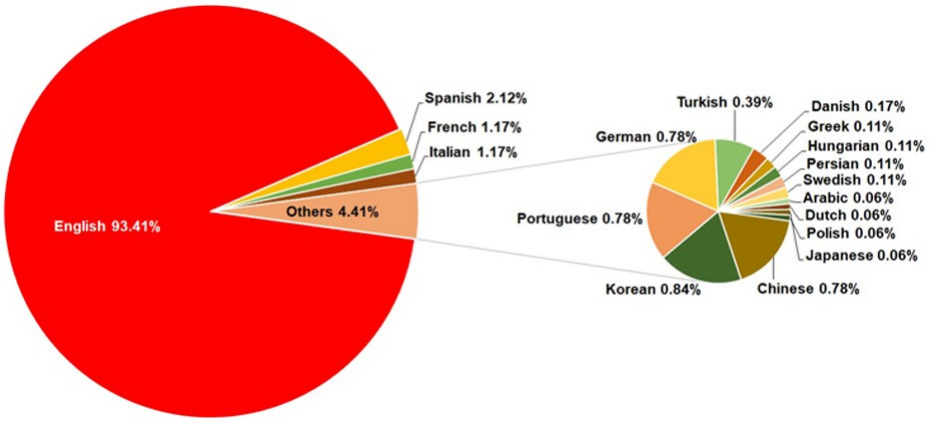
Language of publications on workplace violence in healthcare personnel (1992–2019).

As regards the language of publication, the situation is clear (Fig 1), with English (93.41%) the commonest language of publication, followed by Spanish (2.12%), French (1.17%) and Italian (1.17%). Fig 1 also specifies the less popular languages included in the category "Others" (4.41%).

### Trends of publications and citations

**Fig 2:**
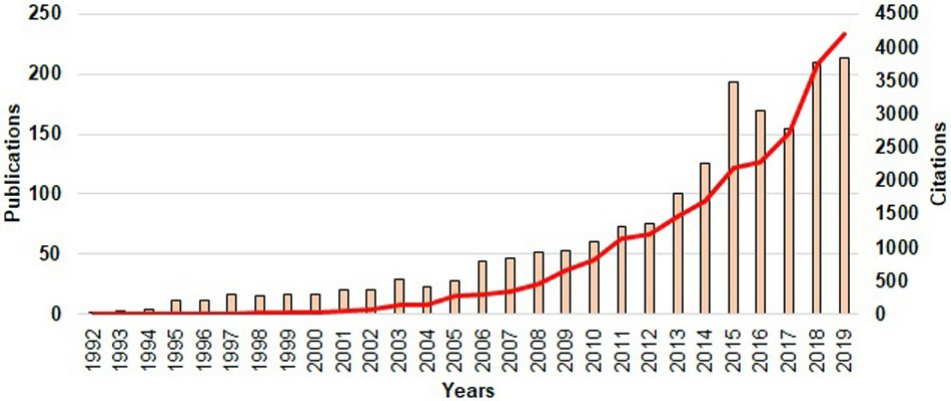
Trends of publications and citations on workplace violence in healthcare personnel (1992–2019). The graph shows annual publications (orange columns) and citations (red line).

As shown in Fig 2, the first article published on this topic dates from 1992. The maximum number of annual publications appears in 2019, with a total of 213. From 2004 onwards, the number of publications rises exponentially, with an R^2^ coefficient close to 0.89 (*p* < 0.0001). This trend, however, is interrupted for two years (2016 and 2017), in which the number of publications was lower than expected.

In addition, documents began to be cited in 1995 and the trend in the number of annual citations increased after this year (R^2^ coefficient = 0.73; *p* < 0.0001). Until 2002, the citations on this topic did not exceed one hundred citations per year. From 2003 (n = 148) and 2004 (n = 150) onwards, there was a considerable rise in the number of citations each year, reaching a peak in 2019 (n = 4,198).

### Most active journals

The top-10 most active journals publishing on this topic from 1992 to 2019 are shown in Table 3. *Journal of Nursing Management* (n = 34), *Journal of Clinical Nursing* (n = 33), *WORK—A Journal of Prevention Assessment & Rehabilitation* (n = 31) and *Workplace Health & Safety* (n = 30) were the only journals which equalled or exceeded a total of 30 documents. In fact, the first two journals belong to the first quartile (Q1 JCR and SJR in 2018) and the last two to the fourth quartile (Q4 JCR in 2018) and second quartile (Q2 SJR in 2018). In general terms, the top-10 journals had high quartiles and IF values. The highest IF belongs to *Lancet* with 59.102 (2018 JCR) and 15.87 (2018 SJR).

**Table 3:** Top-10 journals with the largest number of publications related to workplace violence in healthcare personnel (1992–2019).

Journals	Categories	Number of documents (percentages)	Citations	CPP	Quartile JCR	2018 Journal Impact Factor in JCR	Quartile SJR	2018 Journal Impact Factor in SJR
***Journal of Nursing Management***	Nursing	34 (1.90%)	708	20.82	Q1	2.386	Q1	1.07
***Journal of Clinical Nursing***	Nursing	33 (1.84%)	776	23.52	Q1	1.757	Q1	0.77
***WORK—A Journal of Prevention Assessment & Rehabilitation***	Public, environmental & occupational health	31 (1.73%)	284	9.16	Q4	1.009	Q2	0.53
***Workplace Health & Safety***	Nursing	30 (1.68%)	179	5.97	Q4	0.922	Q2	0.34
***BMJ Open***	Medicine, general & internal	22 (1.23%)	242	11.00	Q2	2.376	Q1	1.32
***Journal of Emergency Nursing***	Emergency medicine / Nursing	21 (1.17%)	311	14.81	Q3 / Q2	1.489	Q2	0.33
***Journal of Nursing Administration***	Nursing	21 (1.17%)	422	20.10	Q3	1.206	Q1	0.66
***Lancet***	Medicine, general & internal	20 (1.12%)	270	13.50	Q1	59.102	Q1	15.87
***Journal of Advanced Nursing***	Nursing	19 (1.06%)	811	42.68	Q1	2.376	Q1	1.01
***International Journal of Nursing Studies***	Nursing	17 (0.95%)	832	48.94	Q1	3.570	Q1	1.56

CPP, citation per publication; JCR, Journal Citation Reports; SJR, SCImago Journal Rank.

In addition, *International Journal of Nursing Studies* received the most citations (n = 832) and had the highest number of CPP (48.94). As can be seen, seven of the ten journals were in the category “*Nursing*”.

### Analysis of authors and papers

The 1791 publications were written by a total of 40,235 different authors. Of the top-10 authors mainly publishing articles, 6 came from the United States and 3 from Australia (Table 4). D. M. Gates was the author with the largest number of publications (21 records), with 9 as the first author. D. Jackson was the most-cited author with 807 citations and average of 57.64 CPP, and was the first author of 4 publications from a total of 14.

**Table 4:** Top-10 authors with the largest number of publications related to workplace violence in healthcare personnel (1992–2019).

Rank	Author	Country	Institution	Number of publications	First	Last	Other	Single author	Number of citations	CPP
**1**	D. M. Gates	United States	University of Cincinnati	21	9	1	9	2	587	27.95
**2**	G. L. Gillespie	United States	University of Cincinnati	15	10	0	5	0	614	40.93
**3**	M. Hutchinson	Australia	Southern Cross University	14	11	0	2	1	591	42.21
**4**	D. Jackson	Australia	University of Technology Sydney	14	4	4	6	0	807	57.64
**5**	M. J. Boyle	Australia	Griffith University	13	6	1	5	1	171	13.15
**6**	S. G. Gerberich	United States	University of Minnesota	12	2	1	8	1	658	54.83
**7**	J. A. Lipscomb	United States	University of Maryland	12	0	8	4	0	140	11.67
**8**	J. E. Arnetz	United States	Michigan State University	11	9	1	1	0	286	26.00
**9**	P. M. McGovern	United States	University of Minnesota	11	0	1	10	0	620	56.36
**10**	H. H. Wang	Taiwan	Kaohsiung Medical University	10	0	3	7	0	46	4.60

**Fig 3:**
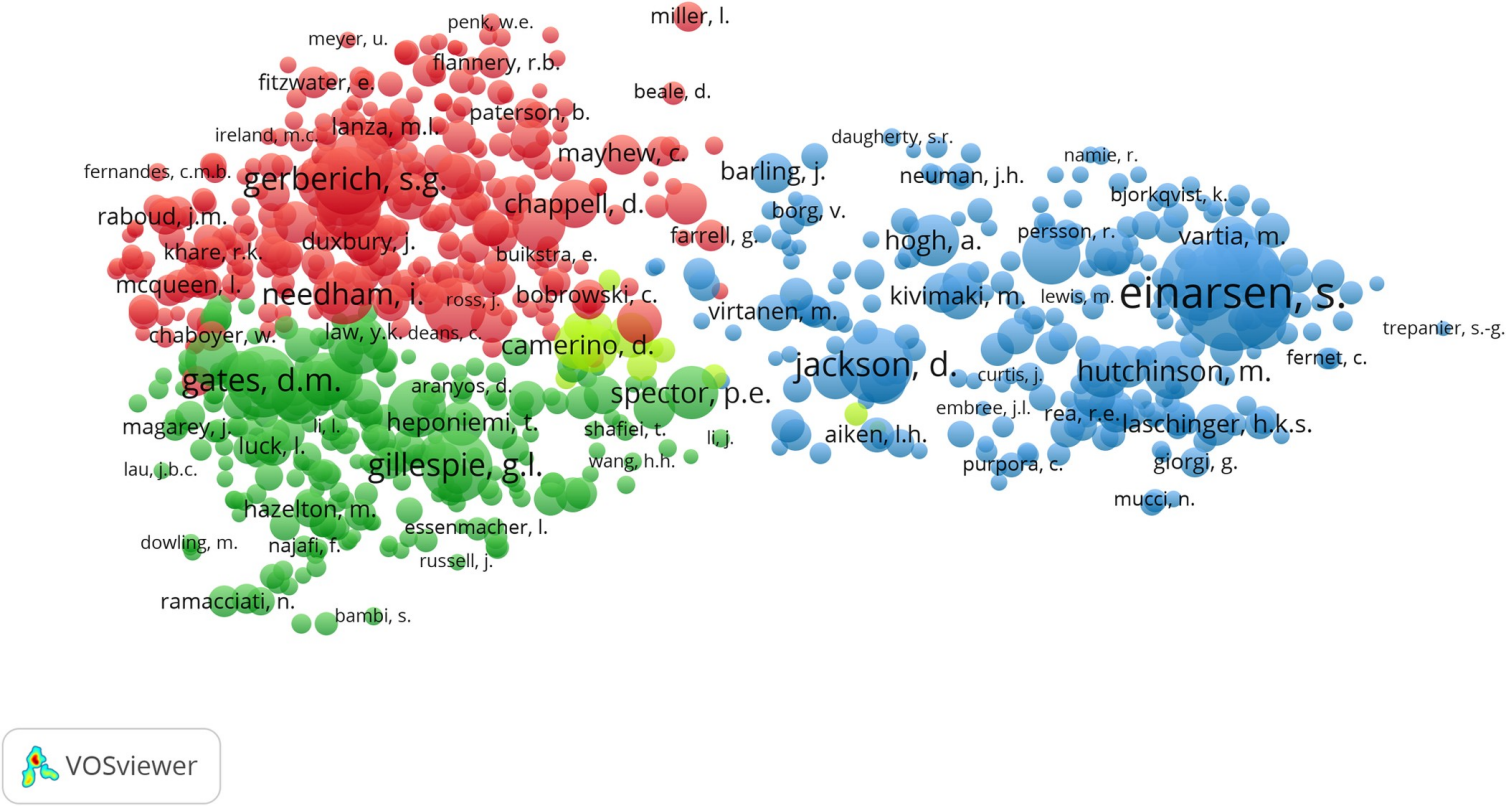
Co-citation map based on cited authors (1992–2019).

The results of the co-citation map are shown in Fig 3. Of 40,235 authors, 896 met the threshold, using 20 as minimum number of citations of an author. Each node represents an author, and its size indicates the number of times the author was referenced in the documents. A link between two nodes indicates a co-citation relationship. Each link has a strength: the thicker the link, the greater the strength of this relationship. The nodes are also grouped according to similarity.

The co-citation map illustrates four different clusters, where each cluster represents a field of this topic: a green cluster (upper left), a red cluster (bottom left), a yellow cluster (in the middle) and a blue cluster (right). The yellow cluster overlaps more with the other clusters. Based on the examination of the titles of all individual papers in the four clusters, a suitable marker was assigned to each of them. The green cluster mainly symbolises violence in healthcare professionals working in emergency department; the red cluster represents publications mainly about nurses; the yellow cluster consists of the European NEXT Study [32], which investigated the working conditions and career prospects of nursing staff in ten European countries; and finally, publications in the blue cluster focused especially on bullying and harassment in the workplace in healthcare organisations.

Table 5 shows the top-10 papers according to the number of citations. The average number of citations was 242.6. Only 3 articles were cited more than 300 times. There were 6 different countries of origin, 9 different journals and 7 different fields in this selection. The vast majority (n = 8) of these articles were published between 2000 and 2010.

**Table 5:** Top-10 most commonly-cited papers related to workplace violence in healthcare personnel research (1992–2019).

Rank	Article title	Author/s	Affiliation	Journal name (JCR Impact Factor 2018)	Published year	Times cited	Field	Field-Weighted Citation Impact
**1**	Workplace bullying in NHS community trust: Staff questionnaire survey	L. Quine	University of Kent, United Kingdom	*British Medical Journal*	1999	344	Medicine	7.89
**2**	Workplace bullying and the risk of cardiovascular disease and depression	M. Kivimäki *et al*.	University of Helsinki, Finland	*Occupational and Environmental Medicine*	2003	332	Public Health, environmental and occupational health / Medicine	5.96
**3**	An epidemiological study of the magnitude and consequences of work related violence: The Minnesota Nurses' Study	S. G. Gerberich *et al*.	University of Minnesota, United States	*Occupational and Environmental Medicine*	2004	304	Public Health, environmental and occupational health / Medicine	4.08
**4**	Who would want to be a nurse? Violence in the workplace—A factor in recruitment and retention	D. Jackson *et al*.	University of Western Sydney, Australia	*Journal of Nursing Management*	2002	235	Leadership and management / Nursing	11.78
**5**	Behind closed doors: in-home workers' experience of sexual harassment and workplace violence	J. Barling *et al*.	Queen's University, Canada	*Journal of Occupational Health Psychology*	2001	227	Health professions / Psychology	3.57
**6**	Workplace bullying in nurses	L. Quine	University of Kent, United Kingdom	*Journal of Health Psychology*	2001	211	Psychology / Applied psychology	0.87
**7**	Violence toward nurses, the work environment, and patient outcomes	M. Roche *et al*.	University of Technology Sydney, Australia	*Journal of Nursing Scholarship*	2010	206	Nursing	13.14
**8**	Violence against nurses and its impact on stress and productivity	D. M. Gates *et al*.	University of Cincinnati, United States	*Nursing Economics*	2011	193	Leadership and management / Nursing	13.63
**9**	Work-related factors and violence among nursing staff in the European NEXT study: A longitudinal cohort study	D. Camerino *et al*.	University of Milan, Italy	*International Journal of Nursing Studies*	2008	190	Nursing	10.04
**10**	Scoping workplace aggression in nursing: Findings from an Australian study	G.A. Farrell *et al*.	La Trobe University, Australia	*Journal of Advanced Nursing*	2006	184	Nursing	5.79

JCR, Journal Citation Reports; Field-weighted citation impact shows how well this document is cited when compared to similar documents. A value greater than 1.00 means the document is more cited than expected.

### Most influential institutions and countries

The University of Cincinnati leads the ranking of the most influential institutions in Scopus in terms of the number of documents (n = 30), closely followed by Monash University (n = 28). In the same way, the institution which obtained the most citations on this topic is the University of Cincinnati (n = 1,111), followed by Western Sydney University (n = 910). Nearly all the top-10 institutions were universities (Table 6).

**Table 6:** Top-10 most influential institutions publishing on workplace violence in healthcare personnel (1992–2019).

Rank	Institutions	Countries	Number of documents	Citations
**1**	University of Cincinnati	United States	30	1111
**2**	Monash University	Australia	28	492
**3**	Harbin Medical University	China	20	320
**4**	Western Sydney University	Australia	19	910
**5**	University of Queensland	Australia	18	294
**6**	Southern Cross University	Australia	17	391
**7**	Karolinska Institutet	Sweden	17	348
**8**	National Institute for Occupational Safety and Health	United States	16	406
**9**	Harvard Medical School	United States	16	142
**10**	University of Newcastle	Australia	15	326

**Fig 4:**
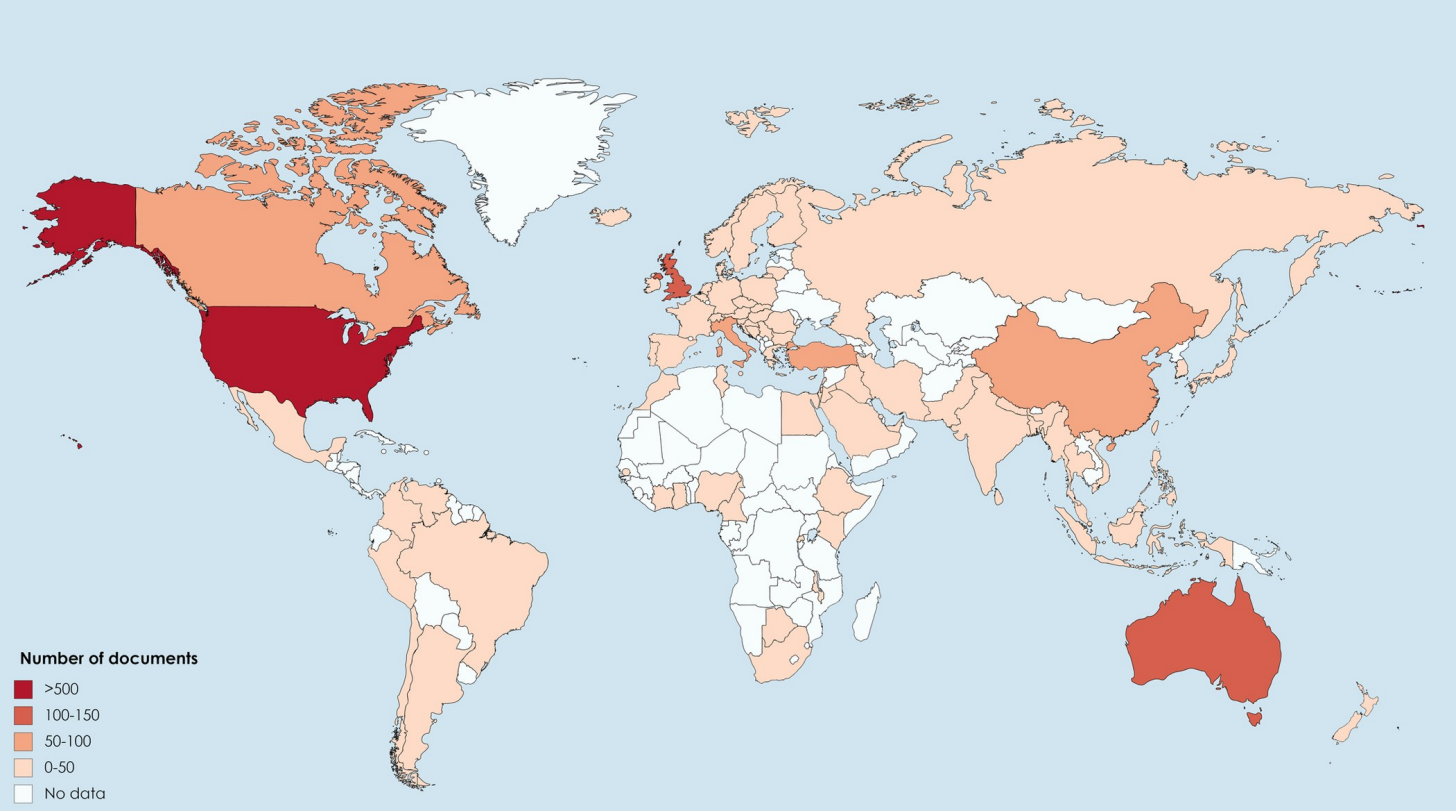
Worldwide distribution of publications on workplace violence in healthcare personnel (1992–2019).

All in all, the publications on workplace violence in healthcare personnel originate from 85 different countries. Fig 4 shows the worldwide distribution of the contributing countries. Thus, United States produced by far the most publications (n = 549); Australia (n = 183) and United Kingdom (n = 110) produced between 100 and 150 publications; 4 countries (4.70%; in decreasing order: Canada, China, Italy and Turkey) produced between 50 and 100 publications and 78 countries (91.76%) produced 50 or less documents. Similarly, documents from United States obtained the most citations (n = 8,068).

**Fig 5:**
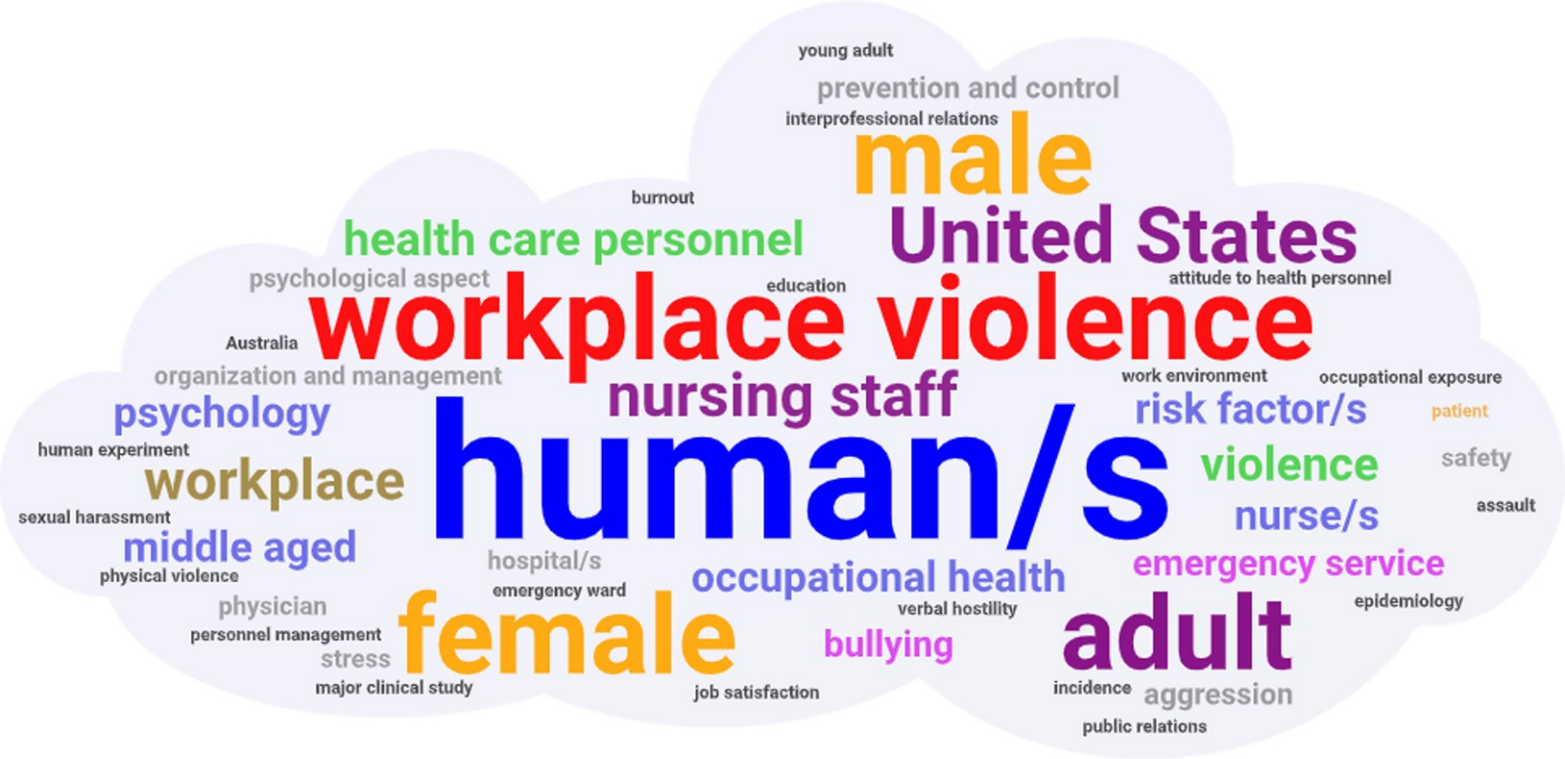
Word cloud of global research keywords about workplace violence in healthcare personnel (1992–2019).

It should be noted that Fig 5 illustrated keywords such as “*human*/*s*” (16.43%), “*workplace violence*” (5.44%), “*female*” (5.06%) and “*male*” (4.79%), which were the most repeated words in publications. It is worth noting that the term “*human*/*s*” was probably used to differentiate from animal research, rather than because of significance to the topic. Furthermore, it is not surprising that terms like "*workplace violence*" (together or separately), "*bullying*", "*emergency service*", "*aggression*" or "*health care personnel*" were present in this topic.

## Discussion

The increase or decrease in the number of scientific publications indicate the speed of scientific/technological development [33]. The present study shows the growth in documents published worldwide on workplace violence in health professionals between 1992 and 2019. This growth in publications may means this research area is being of continuous concern [34]. In the same way, the increasing citation trend in this field further show how workplace violence committed by patients or visitors is present towards health workers globally [35]. Furthermore, the majority of document type published was journal articles, because articles are used as a popular means to advance the development of a specific knowledge in a research area [36]. In addition, new articles and numerous citations may be related to the importance of an issue to the general public and publication policy [37, 38].

Numerous publications have tended to focus on nurses, physicians or emergency medical service personnel [39–42]. This fact might explain the commonest subject category of documents on workplace violence in health professionals was, by far, medicine, followed by nursing. A range of 8–38% of healthcare workers worldwide reported some manners of violence at some point in their careers [14]. Nevertheless, a recent systematic review and meta-analysis reported a 61.9%, being nurses (59.2%) and physicians (56.8%) were more often the target of workplace violence than other healthcare professionals (44.4%) [35].

As regards countries, United States is clearly the most productive country in terms of document output about this topic, followed by Australia and United Kingdom. In the same manner, University of Cincinnati of United States and Monash University of Australia are the two leading institutions, followed by Harbin Medical University of China and Western Sydney University of Australia. These results are in line with the prevalence of workplace violence in health personnel in Australia (70.9%), North America (67.3%), Asia (64.9%), Africa (59.2%) and Europe (48.1%) [35]. Therefore, it is likely that institutions in these countries want to understand this phenomenon especially, try to study its implications for the quality of care and the well-being of the workers and determine preventive and punitive measures that should be employed to diminish the occurrence of workplace violence in health professionals [43]. Other possible reason might be the traditional culture difference, funding input, and economic level. Another possible reason is that while the Scopus database is comprehensive, some journals published from other east regions are not indexed in Scopus. Furthermore, as most scientific literature is published in English, some non-native English-speaking researchers might not produce high quality papers due to the language problem to some extent. These thoughts might explain the low productivity from east countries. In the same way, the majority of top-10 authors were from United States or Australia. As found in other research areas, collaborative regions, institutions and authors ten to be geographically correlated [44]. Moreover, the results of co-citation analysis of highly-cited authors showed clearly four distinct clusters, which represented a subfield of this research area. These results support the idea of a high number of co-citations in published material indicates a closer relationship among the authors within the same subfield and an opportunity for future collaboration [45]. In addition, the goal of workplace violence risk assessment is not only to predict violence, but instead to identify and prioritize concerning aspects of a given scenario and translate findings into management strategies [46].

Bibliometrics analysis is based on the utilization of different measures/indicators [47], such as the ISI Impact Factor [48] by JCR [49] or the SJR [50] by SCImago Journal and Country Rank (SJC) [51]. In that sense, at least half of top-10 scientific journals which focus on workplace violence in healthcare personnel were in the highest quartile score (Q1) both JCR and SJR. Although these impact factors have been used to evaluate the quality of scientific material published in journals [52], its score has been questioned [53]. It is essential that last decades has seen an increasing number of documents globally of this field of research, due to authorities and general population have attested to significant worry at this public health problem [54]. Therefore, researchers prefer publish studies in high impact journals and gain visibility [55]. For its part, the first two top-10 papers most cited were published in 1995 and 1999, respectively. The publication more recent in this top was published in 2011. It should be noted that the times of citation in a document is highly correlated with the date of that publication, being older publications sometimes more cited than newer publications [56, 57]. For its part, most active journals publishing on workplace violence in healthcare personnel were nursing journals. This could be because nurses have more opportunities to deal with patients and their families than physicians do in day-to-day clinical encounters. Therefore, this places frontline this healthcare provider group at an especially high risk of workplace violence [54].

Nowadays, the language that most researchers who read and publish is English [58, 59]. This finding was in line in present study because English was the commonest language of publication. This is due to articles were more visible and cite by the scientific community and are accessible to a larger audience [60].

At last, the world cloud showed the most common keywords was clearly "*human*/*s*". It is essential to highlight that this keyword could be used as a heuristic that the document was about clinical research [61].

As regards the limitations of this study, it should be noted that there may be studies of workplace violence in health professionals that have been published in other databases and that not all published records have the same proportion of scientific knowledge.

Overall, the present manuscript adds to the literature by elucidating the growing concern of this public health problem. This study can help potential researchers to quickly understand workplace violence against healthcare professionals globally. It also can provide useful information for relevant research in terms of identifying the research trends and potential collaborators. Additionally, this study can help policy makers improve policy making to prevent workplace violence.

## Conclusions

From 1992 to 2019, worldwide research into the published literature on workplace violence in healthcare personnel has grown steadily year by year, both in the number of documents and the number of citations. In this scientific literature, English is the predominant language, the journal article is the most popular format and the most frequent subject category is “*Medicine*”. In addition, the University of Cincinnati (United States) was the institution (and country) with the highest number of publications, with D. M. Gates heading the list of the top authors. *Journal of Nursing Management* was the favourite journal for publishing and the commonest keyword was “*human*/*s*”.

## Supporting information

S1 Dataset Research data about workplace violence in healthcare personnel using the Scopus database. (RAR)
